# Cardiovascular Health Equity for Survivors of Childhood Cancer: Understanding the Drivers of Cardiovascular Risk∗

**DOI:** 10.1016/j.jaccao.2023.05.010

**Published:** 2023-08-15

**Authors:** Garth Graham, Viknesh Sounderajah, Michelle N. Johnson

**Affiliations:** a Google, San Bruno, California, USA; b Institute of Global Health Innovation, Imperial College London, London, United Kingdom; c Memorial Sloan Kettering, New York, New York, USA

**Keywords:** cancer survivorship, health disparities, health equity, race/ethnicity, social determinants of health

**Figure:**
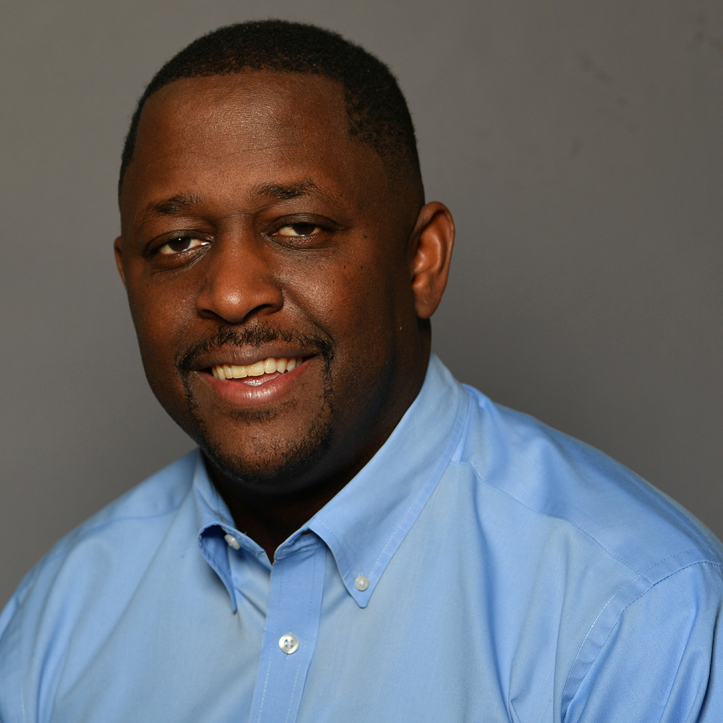

Advances in the treatment of childhood cancer have seen a laudable increase in the number of survivors who live into adulthood.^1^ However, the impact upon the long-term health of these individuals remains sizable.^2^ Within this cohort, cardiac disease has emerged as the most common cause of late mortality, second only to further malignancy.^3^ This is due in large part to the long-term cardiotoxicity that is associated with the use of intensive chemotherapy regimens and radiotherapy. These issues manifest as a broad spectrum of cardiac conditions, including coronary artery disease, arrhythmia, valvular disease, and heart failure.^4^ Exposure to these hazards is seen as largely nonmodifiable given the necessity of these treatments. Despite this, there is some encouraging evidence to suggest that modifications to, and judicious use of, contemporary oncological treatment strategies,^5^ such as more conservative regimes of chest radiotherapy and anthracyclines, have been associated with a reduced risk for coronary artery disease among survivors.^6^ Unfortunately, similarly close attention has not been paid to the diagnosis and treatment of modifiable cardiovascular risk factors^7^ (CVRFs) within this cohort. Issues such as hypertension, dyslipidemia, and glucose intolerance or diabetes compound the already grave cardiovascular burden this vulnerable population group faces and significantly increase their overall risk for cardiac events. Worse still, it is suggested that childhood cancer survivors can be up to twice as likely to be undertreated^7^ for these conditions in comparison with the general population. This is a particularly worrying statistic for Black and Hispanic population groups, which carry a disproportionately high burden of such risk factors because of the complex interplay among the individual and societal determinants of health^8^ to which they are typically exposed. However, what has remained unknown until now is whether a history of childhood cancer modifies the typical incidence of CVRF disparities that is established across racial/ethnic groups in the general population.

In this issue of *JACC: CardioOncology*, a study reported by Noyd et al^9^ addresses this gap in the literature by investigating whether disparities in CVRFs by race/ethnicity are similar among childhood cancer survivors compared with the general population. To achieve this aim, the investigators leveraged 3 specific datasets: the retrospective CCSS (Childhood Cancer Survivor Study) cohort, which served as the population of interest, in addition to the CCSS sibling cohort and data from NHANES (National Health and Nutrition Examination Survey), which both served as the “general population” comparator cohorts. The rationale for using 2 comparison cohorts was to leverage sibling control subjects and to consider a sample of adults from the general population.

Of the 16,457 survivors eligible for analysis from the initial CCSS cohort of 25,656 who completed baseline questionnaires, 3 groups stratified by race/ethnicity were formed: non-Hispanic White (NHW) (n = 13,960), non-Hispanic Black (NHB) (n = 1,092), and Hispanic (n = 1,405). Within these groups, NHB and Hispanic survivors reported a higher cumulative incidence of diabetes, obesity, multiple CVRFs, and, for NHB survivors, hypertension by 40 years of age in comparison with NHW survivors. Moreover, when controlling for sociodemographic and treatment factors compared with NHW survivors, the incidence rate ratios (IRR) for NHB were increased for hypertension, obesity, and multiple CVRFs, whereas for Hispanic survivors, the IRR was increased for diabetes and obesity. Of note, the pattern of IRRs for CVRF differences was similar among CCSS sibling and NHANES cohorts, respectively. This would suggest that differences in the incidence of CVRFs on the basis of race/ethnicity among survivors were similar to those observed in the general population, which addresses the primary objective of the study.

As a minimum, these findings should serve to catalyze the development and implementation of equitable survivorship-focused care programs nationally.^10^ As Noyd et al^9^ note, childhood cancer survivors, despite sharing similar oncological diagnoses, represent an ethnically and sociodemographically diverse group. As such, there is no one-size-fits-all model in how such services are designed and delivered. At-risk populations necessitate stratified and personalized plans with respect to the prevention, surveillance, and treatment of their cardiovascular health. Currently, health care professionals who wish to deliver such a service do not have access to specific guidance in how best to manage survivors as stratified by ethnicity/race.^11^ To ensure consistency in the delivery of such a program, surveillance and treatment tenets must be codified in national-level guidance, ideally through the formation of consensus derived scientific recommendations.

Although the design and delivery of such a service would represent an important step in the right direction, there is also much work to be done in understanding the specific drivers of the observed disparities. As noted in the study, differences by race/ethnicity persisted despite adjustment for individual-level socioeconomic factors. Despite this statement, it is crucial not to surmise that social determinants of health are unrelated to what has been observed. As highlighted by Churchwell et al,^12^ health disparities are fundamentally driven by structural racism and other forms of societal biases. Many of these biases are not adequately captured with existing datasets or instruments, which leads to gaps in data, understanding, and, ultimately, progress.

As such, we should look to challenge how data on determinants of health are collected for such large, multiyear observational studies, which typically form the foundation of multiple analyses and studies. In addition to greater individual-level data granularity (with reduced reliance upon self-reported outcome measures, which introduce detection bias), it is essential to routinely capture robust area-level measures. Encouragingly, for future initiatives, this once persistent data gap can be addressed by the Agency for Healthcare Research and Quality’s initiative to establish a Social Determinants of Health Database,^13^ whose aim is to provide vital data regarding social context, economic context, education, physical infrastructure, and health care context; the 5 key domains that constitute the social determinants of health model.^14^ Further investigation leveraging such rich area-level data could highlight trends that are not apparent in individual-level data and may serve to underpin the development of location-specific interventions.

However, despite the limitations conferred by the available data, the investigators are to be commended for providing a call to arms for health care professionals who are invested in the long-term care of childhood cancer survivors. As this cohort ages, almost 75% of survivors will have at least 1 chronic health condition, and >40% will have serious or life-threatening conditions by 30 years after their cancer diagnoses.^15^ Through the findings of this study, we now have empirical evidence to suggest that minority groups, which already face daily societal challenges engendered through deep-seated structural biases, also face the steepest cardiovascular survivorship burden. Knowing this, it is our duty to drive survivorship program reform to ensure that fair and equitable cardiovascular care is delivered to all.

## Funding Support and Author Disclosures

The authors have reported that they have no relationships relevant to the contents of this paper to disclose.
